# Notes and News

**Published:** 1901-06-22

**Authors:** 


### Notes and News.

Several fresh cases of plague are reported from Zagazig, Cairo.

A new ward has been added to the Passmore Edwards District Hospital at Tilbury.

The epidemic of plague in Hong Kong is very severe, the last return we have notifies the number of deaths in week as being equal to the number of new cases.

The disputed monetary arrangement between St. Bartholomew's Hospital and the Almoners of Christ's Hospital respecting the site to be vacated by the latter, has been referred to arbitration.

Messrs. J. S. Morgan and Co. have sent a cheque for £2,650 from Mr. Foxhall P. Keene to the Prince of Wales's Hospital Fund, being one half of the stakes won by Mr. Keene's horse at the Oaks.

The Chelsea Hospital for Women has received £127 13s. collected by Mrs. Sington, and £52 10s. from T. W. Brookes, Esq., J.P., towards the reflooring of its wards advocated by the Prince of Wales's Hospital Fund.

The Men's Convalescent Home at Iyn-y-coed, in connection with the Birmingham Hospital Fund, has just been extended. Miss Stokes, who in conjunction with her late sister, founded the home, is now defraying the whole cost of the extension, amounting to upwards of £5,000. Miss Stokes and her sister have given more than £16,000 in all to the institution.

The Association for the Oral Instruction of the Deaf and Dumb is doing a most admirable work, but it is sadly crippled for want of funds. The Association has its own school, regularly reported upon by Government inspectors, and training is given to teachers in oral and lip reading methods of instructing. To those who are unacquainted with the truly remarkable methods of opening the outer world to the deaf and dumb a visit to the training college on a Wednesday afternoon at three o'clock, when a demonstration lesson is given, will be found of great interest.

A short time back a new Eye and Ear Hospital was opened at Tunbridge Wells. The institution, which is a dwelling-house adapted to hospital purposes, has been thoroughly re-arranged and decorated.

Provision is to be made at the Newhills Convalescent Home, near Aberdeen, for the outdoor treatment of consumptives. The new wing will accommodate sixteen patients, and be built from foreign designs.

By the will of Mr. Joseph Goodrich, a farmer of Cotton, the East Suffolk and Ipswich and the Bury and West Suffolk Hospitals will benefit to a large extent, the former probably receiving about £30,000 and the latter £10,000.

The Honorary Degree of Doctor of Law has been conferred upon Miss Weston, of the Royal Sailors' Rest, by the University of Glasgow, at the celebration of their 450th Anniversary, in token of their warm appreciation of the great work carried on by herself and her coadjutors in the British Navy.

The cottage hospital at Blairgowrie, N.B., formally opened last month, has at present six beds and one cot; there is a special ward also, not yet fitted up. There is accommodation for more beds in the two large wards if required. The hospital has been open for the reception of patients for two months.

The foundation stone of the new union infirmary for Richmond has been laid. It will consist of six blocks for 172 patients. The new infirmary will provide proper accommodation for carrying out the outdoor treatment of consumption. It is expected that the buildings will be completed next year. The cost is estimated at £40,000. The architect is Mr. Edward J. Partridge.

Extensive and necessary improvements are to be made at the Taunton and Somerset Hospital, the estimated cost of which will be about £6,000. Towards this generous promises have been made by Viscount Portman and the Hon. E. Portman of £1,000 each, of £500 each by Mr. W. Proctor Baker and the Hon. Seymour Portman, and of handsome sums by other friends of the institution.

The Paddington Green Children's Hospital has been obliged to give up its convalescent home at Wealdstone. This is a great loss, and another temporary home will be provided as soon as possible. The committee are most anxious to build a permanent home for the Hospital to house about twenty children. We sincerely hope they will soon find funds forthcoming for so useful and desirable an object.

Mr. Beerbohm Tree has forwarded to the honorary secretaries of the Prince of Wales's Hospital Fund for London at the Bank of England a cheque for £257 11s. being £174 7s. 8d. net proceeds of a matinée given by him and Mrs. Beerbohm Tree at Her Majesty's Theatre on May 2nd in aid of the fund, and £83 3s. 4d., which is a contribution from him and Mrs. Tree "in recognition of the splendid institution for which they have had the honour to work."

